# Study to find the best extraction solvent for use with guava leaves (*Psidium guajava* L.) for high antioxidant efficacy

**DOI:** 10.1002/fsn3.91

**Published:** 2014-02-12

**Authors:** Jongkwon Seo, Soojung Lee, Marcus L Elam, Sarah A Johnson, Jonghoon Kang, Bahram H Arjmandi

**Affiliations:** 1 Department of Nutrition, Food and Exercise Sciences, College of Human Sciences, Florida State UniversityTallahassee, Florida, 32306; 2 Department of Food and Nutrition, Institute of Agriculture and Life Science, Gyeongsang National University501 Jinjudaero, Jinju, 660-701, Korea; 3 Department of Biology, Valdosta State UniversityValdosta, Georgia, 31698

**Keywords:** Antioxidant, flavonoid, guava, hydroethanolic solvent, phenolic compound

## Abstract

The effects of guava leaves extracted using solvents of water, ethanol, methanol, and different concentrations of hydroethanolic solvents on phenolic compounds and flavonoids, and antioxidant properties have been investigated. The antioxidant capability was assessed based on 2,2-diphenyl-1-picrylhydrazyl radical and 2,2′-azinobis-(3-ethylbenzothiazoline-6-sulfonic acid) radical-scavenging abilities, reducing power, and nitric oxide-and nitrate-scavenging activities. The results demonstrated that the antioxidant ability of guava leaf extracts has a strong relationship with phenolic compound content rather than flavonoid content. Phenolic compound content of water extracted guava leaves was higher compared to pure ethanol and methanol extracts. However, phenolic compound content extracted using hydroethanolic solvent was higher than water, whereas 50% hydroethanolic was observed to be the most effective solvent showing high antioxidant ability.

## Introduction

Medicinal plants have been used in the treatment and improvement of human diseases (Gutierrez et al. 2008; Nyirenda et al. 2012), and such plants with high antioxidant abilities can be used as natural medicines for preventing aging and chronic diseases (Kähkönen et al. 1999). In addition, these plants have various physiologically active substances with anticancer and antimicrobial abilities (Bhanot et al. 2011; Miyake and Hiramitsu 2011). The free radical-scavenging abilities of plants has been evaluated by in vitro models of scavenging activities against 2,2-diphenyl-1-picrylhydrazyl (DPPH), 2,2′-azinobis-(3-ethylbenzothiazoline-6-sulfonic acid) (ABTS), superoxide, hydroxyl radical, and nitric oxide radical, reducing power, lipid peroxidation levels, and antioxidant enzyme activities (Brand-Williams et al. 1995; Jayanthi and Lalitha 2011; Reddy et al. 2012). Reactive oxygen species (ROS), such as hydroxyl radical (·OH), superoxide anion (·O^2−^), and hydrogen peroxide (H_2_O_2_), which are produced in the cell system, are known to cause oxidative damage. This damage may cause cellular injuries and exacerbate several degenerative diseases associated with aging, cancer, and cardiovascular disease (Pham-Huy et al. 2008; Sharma and Singh 2012).

Guava (*Psidium guajava* L.), which is used as a traditional medicine, is found in countries with hot climates in areas such as South America, Europe, Africa, and Asia (Gutierrez et al. 2008). Its primary traditional uses include the alleviation of diarrhea and dehydration. Other reported uses include treatment of gastroenteritis, dysentery, stomach pain, diabetes mellitus, and wounds. In addition, it is known for its antioxidant, antibacterial, and anti-inflammatory properties (Qian and Nihorimbere 2004; Cheng et al. 2009; Han et al. 2011a). Guava leaves have phenolic compounds and flavonoids with high antioxidant activity. The main active substances in guava leaves are gallic acid, caffeic acid, guaijaverin (Gutierrez et al. 2008), tannins (Okuda et al. 1987), carotenoids (Mercadante et al. 1999), and triterpenoids (Shao et al. 2012). These substances have been extracted by using several solvents such as water (Moreno et al. 2000), ethanol, hydroethanol (Qian and Nihorimbere 2004), methanol (Chah et al. 2006), and hydromethanol (Bushra et al. 2012). However, there is a paucity of research investigating the most effective solvent for the antioxidant efficacy of guava leaves.

Therefore, in this study, the phenolic compound and flavonoid content of water, ethanol, methanol, and hydroethanolic extracts of guava leaves were analyzed and evaluated with regard to antioxidant properties. The best extraction solvent for use with guava leaves for high antioxidant efficacy was selected.

## Material and Methods

### Chemicals and reagents

Ethanol and methanol were purchased from Duksan (Jinju, Korea). Folin–Ciocalteu reagent, caffeic acid, quercetin, DPPH, ABTS, potassium ferricyanide, trichloroacetic acid, ferric chloride, sulfanilamide, phosphoric acid, and *N*-(1-naphthyl) ethylenediamide were purchased from Sigma (St. Louis, MO). Potassium acetate, sulfanilic acid, and naphthylamine were purchased from Yakuri (Osaka, Japan). All chemicals and reagents were of analytical grade. Guava leaves were obtained from Guava Korea Ltd. (Uiryeong-gun, Korea).

### Preparation of water extracts

As described by Kandil et al. (1994), a sample of 100 g guava leaves in 1.5 L distilled water was boiled for 4 h. The sample was then filtered using Whatman filter paper No. 4. The filtrate was concentrated in a rotary evaporator at 60°C and dried using a freeze drier. The resulting extracts were stored at −18°C until the analysis.

### Preparation of ethanol, methanol, and hydroethanolic extracts

The ethanol and methanol extracts were prepared by placing a sample of 100 g of guava leaves in 1.5 L pure ethanol (purity 94.0%) and 1.5 L pure methanol (purity 99.8%), respectively, for 4 days at room temperature. For the hydroethanolic extracts, hydroethanol solvents with water:ethanol in the ratios of 70:30, 50:50, 30:70, and 10:90 (v/v) were prepared for use in the extraction. After 4 days, the extracts were filtered using Whatman filter paper No. 4, and then the filtrates were concentrated using a rotary evaporator at 50°C. The resulting filtrates were dried using a freeze drier and stored at −18°C until further analysis.

### Phenolic compound content assay

The Folin–Ciocalteu method (Ainsworth and Gillespie 2007) with a modification was used to determine the phenolic compound content of the samples. One milliliter of each extract was diluted with 2 mL distilled water and 0.5 mL of Folin–Ciocalteu reagent (Sigma Co.). After 3 min, 0.5 mL of 10% Na_2_CO_3_ solution was added to the mixture and the mixture was allowed to stand for 1 h at room temperature in a dark room. The absorbance was measured at 760 nm with a UV–visible spectrophotometer (Optizen 2120 UV; Mecasys Co., Ltd., Daejeon, Korea). A standard caffeic acid (Sigma Co.) solution (10–100 *μ*g/mL) was used for the construction of a calibration curve. Results were expressed as mg caffeic acid/g extract. The tests were run in triplicate and averaged.

### Flavonoid content assay

Flavonoid content was determined by Moreno's method (Moreno et al. 2000). Each extract (1 mL) was added to a test tube containing 0.1 mL of 10% aluminum nitrate, 0.1 mL of 1 mol/L aqueous potassium acetate, and 4.3 mL of 80% ethanol. After 40 min at room temperature in a dark room, the absorbance was measured at 415 nm. Total flavonoid content was assessed using quercetin (Sigma Co.) as a standard (0–100 *μ*g/mL).

### 2,2-Diphenyl-1-picrylhydrazyl radical (DPPH^.^)-scavenging assay

A series of water, ethanol, methanol, and hydroethanol guava leaf extracts (50, 100, 250, 500 and 1000 *μ*g/mL) were prepared for an antioxidant assay. Scavenging activity of the extracts on DPPH^.^ was measured according to the method developed by Blois (1958). Varying concentrations of the guava leaf extract solutions (1 mL) were added to a DPPH^.^ methanol solution (5 mg/100 mL, 2 mL). The decrease in absorbance at 517 nm was measured with a UV–visible spectrophotometer. DPPH^.^-scavenging activity (%) was calculated according to the following equation:


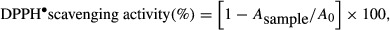
(1)

where *A*_sample_ is the absorbance of the sample solution in a steady state and *A*_0_ is the absorbance of DPPH^.^ solution before adding the extract.

### Scavenging activity on ABTS^.+^

ABTS^.+^-scavenging activity was assessed according to the method described by Re et al. (1999). A mixture of ABTS (7.0 mmol/L) and potassium persulfate (2.45 mmol/L) in water was prepared and stored at room temperature for 12 h in a dark room to produce ABTS^.+^. The ABTS^.+^ solution in water was diluted to the level of absorbance of 1.50 at 414 nm for the analysis. Different concentrations of the extract solution (1 mL) were added to the diluted ABTS^.+^ solution (2 mL). The absorbance was recorded at 414 nm. The radical-scavenging activity was measured according to equation (1).

### Reducing power assay

The reducing power was measured by the browning reaction method (Oyaizu 1986). Varying concentrations of the extract solutions (1.0 mL) were mixed with phosphate buffer (pH 6.6, 1.0 mL, 0.2 mol/L) and 1% aqueous potassium ferricyanide (1.0 mL). The mixture was incubated for 20 min at 50°C. An aliquot (1.0 mL) of 10% aqueous trichloroacetic acid was added to the mixture, which was subsequently centrifuged for 10 min at 5000 rpm. The upper layer of the solution (1.0 mL) was mixed with pure water (1.0 mL) and 0.1% aqueous FeCl_3_ (1.0 mL), and the absorbance was measured at 700 nm.

### Nitric oxide radical-scavenging activity

Nitric oxide radical (NO^.^)-scavenging activity was measured by the Greiss reagent as described in a previous study (Sumanont et al. 2004). Sodium nitroprusside (5 mmol/L) was dissolved in phosphate buffer (pH 7.4, 2 mL), mixed with flavonoid solution (1 mL), and incubated at 25°C for 150 min. The Greiss reagent (0.5 mL) consisted of 2% sulfanilamide in 4% aqueous H_3_PO_4_, and 0.1% aqueous *N*-(1-naphthyl) ethylenediamide (1:1, v/v) was added to the sample solutions. The absorbance was measured at 542 nm. The percentage of scavenging activity was calculated according to equation (1).

### Nitrite-scavenging activity

Nitrite-scavenging activity was evaluated based on the absorbance at 520 nm using a UV-spectrophotometer according to the method reported by Kato et al. (1987). One milliliter of 1 mmol/L NaNO_2_ (Sigma Co.) solution was added to 1 mL of each sample, and the resulting mixtures were adjusted to pH 2.5 using 0.1 N HCl and 0.2 N citric acid solutions. Each sample was allowed to react at 37°C for 1 h, after which 1 mL of each sample was taken from the solution and mixed thoroughly with 3 mL of 2% acetic acid and 0.4 mL of the Griess reagent. The solutions were stored at room temperature for 15 min. The Griess reagent was prepared by mixing an equal amount of 1% sulfanilic acid (Sigma Co.) and 1% naphthylamine (Sigma Co.), which were made with 3% acetic acid. Nitrite-scavenging activity was calculated according to equation (1).

### Statistical analysis

All experiments were carried out in triplicate. Values are presented as mean ± SD (*n* = 3). Statistical differences among the groups were determined by analysis of variance followed by Duncan's multiple range test using the SPSS program (version 12.0; SPSS Inc., Chicago, IL) package. *P *<* *0.05 was considered statistically significant.

## Results and Discussion

### Phenolic compound and flavonoid content

**Figure 1:**
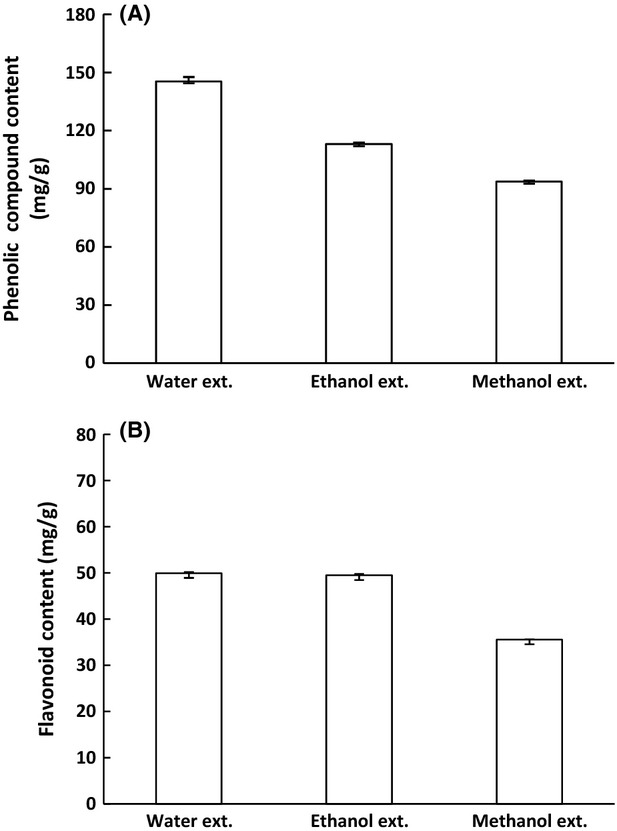
Phenolic compound and flavonoid content of guava leaf extracts for each extract solvent. Phenolic compound content of guava leaf extract (A), Flavonoid content of guava leaf extract (B). The results are expressed as mean ± SD. The significance of differences was determined by one-way analysis of variance using SPSS version 12.0. A *P *<* *0.05 indicates that the difference is significant.

**Figure 2:**
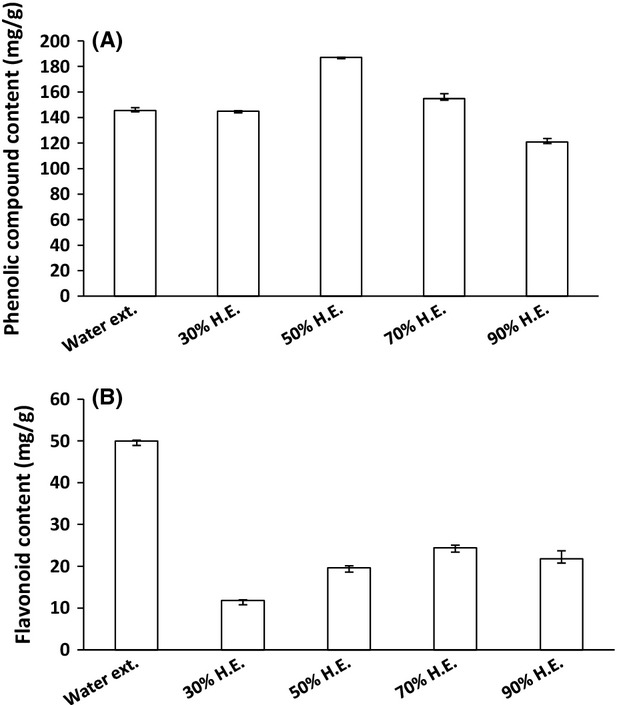
Phenolic compound and flavonoid content of guava leaf extracts for each concentration of hydroethanolic solvent. Phenolic compound content of guava leaf hydroethanolic extracts (A), flavonoid content of guava leaf hydroethanolic extracts (B). H.E., hydroethanolic extract. The results are expressed as mean ± SD. The significance of differences was determined by one-way analysis of variance using SPSS version 12.0. A *P *<* *0.05 indicates that the difference is significant.

Total phenolic compound and flavonoid content of guava leaf extracts are listed in Figures 1, 2. The phenolic compound content of water extract was higher than that of the pure ethanol and pure methanol extracts (Fig. 1A). Furthermore, the phenolic compound content of the hydrophenolic extracts was higher than that of the water extract, and the highest content of phenolic compounds was in the 50% hydroethanolic extract (Fig. 2A). Among the three solvent extracts, the flavonoid content of the water and ethanol extracts was higher than that of the methanol extract (Fig. 1B). Among the four concentrations of the hydroethanolic extracts, the flavonoid content of the 70% hydroethanolic extract was the highest (Fig. 2B).

This result is consistent with previous reports showing that the phenolic compound content of water extract was higher than in pure ethanol and pure methanol extracts (Reddy et al. 2012; Aktumsek et al. 2013). Nyirenda et al. (2012) reported that polar compounds, such as phenolic compounds and flavonoids, were more soluble in aqueous solvents than in organic solvents. It was reported that the phenolic compound content of 50% hydroethanolic extract was higher than in the water extract of guava leaves (Qian and Nihorimbere 2004). Another study found that the order of increasing phenolic compound content of *Hieracium pilosella* was 50% hydroethanolic extract > 80% hydromethanolic extract > water extract (Stanojević et al. 2009). According to another research study, the phenolic compound content was highest in 40% hydroethanolic extract (Ito et al. 2012). Our results agree with several studies that examined the relationship between phenolic compounds and antioxidant capacity. A previous study found that antioxidant capacity varied according to the phenolic compound profile (Kosińska et al. 2012). Another study reported that there are positive correlations between the phenolic compound concentration and antioxidant ability (Kim et al., 2008).

### DPPH-^.^and ABTS^.+^-scavenging activity and reducing power

The antioxidant properties of three solvent extracts were evaluated by in vitro tests including DPPH-^.^ and ABTS^.+^-scavenging activity and reducing power (Table 1). In all measurements, the antioxidant capacity was observed to be significantly higher in the water extract that had the highest content of phenolic compounds, which suggests a positive correlation between the antioxidant capacity and the phenolic compound content. The antioxidant activities increased depending on the concentration of the extracts. Furthermore, the antioxidant activity of the hydroethanolic extracts was higher than that of the water extracts and was highest for 50% hydroethanolic extract (Table 2).

**Table 1:** Antioxidant activities of guava leaf extracts for each of the three extract solvents.

	Solvents	Sample concentration (*μ*g/mL)				
		50	100	250	500	1000
DPPH	Water	28.12 ± 0.21^aB^	51.51 ± 1.09^bC^	89.00 ± 0.52^cC^	92.79 ± 0.15^dB^	93.86 ± 0.06^eB^
	Ethanol	18.97 ± 1.66^aA^	35.57 ± 2.87^bB^	71.80 ± 0.53^cB^	92.78 ± 0.35^dB^	92.95 ± 0.08^dB^
	Methanol	18.76 ± 3.58^aA^	24.33 ± 1.20^bA^	49.88 ± 1.63^cA^	88.07 ± 2.22^dA^	90.29 ± 2.05^dA^
ABTS	Water	37.17 ± 0.37^aC^	64.27 ± 0.23^bC^	97.18 ± 0.00^cC^	98.29 ± 0.13^dC^	98.74 ± 0.07^eC^
	Ethanol	21.12 ± 0.38^aB^	41.05 ± 3.77^bB^	81.01 ± 1.12^cB^	91.27 ± 0.26^dB^	94.26 ± 0.19^dB^
	Methanol	16.25 ± 2.87^aA^	25.89 ± 3.73^bA^	50.17 ± 3.48^cA^	82.22 ± 1.89^dA^	85.09 ± 0.27^dA^
Reducing power	Water	0.19 ± 0.00^aC^	0.28 ± 0.00^bC^	0.51 ± 0.01^cC^	0.83 ± 0.01^dC^	1.35 ± 0.00^eC^
	Ethanol	0.12 ± 0.01^aA^	0.16 ± 0.01^bA^	0.24 ± 0.00^cA^	0.40 ± 0.00^dA^	0.69 ± 0.02^eA^
	Methanol	0.16 ± 0.00^aB^	0.21 ± 0.01^bB^	0.40 ± 0.01^cB^	0.67 ± 0.03^dB^	1.15 ± 0.02^eB^
NO	Water	12.54 ± 1.42^aA^	14.45 ± 2.30^abA^	18.05 ± 2.52^bA^	27.32 ± 2.76^cA^	35.20 ± 2.13^dA^
	Ethanol	27.29 ± 0.71^aB^	34.62 ± 0.37^bC^	36.22 ± 2.32^bC^	39.76 ± 0.09^cC^	41.67 ± 0.65^cB^
	Methanol	25.33 ± 1.89^aB^	28.63 ± 1.47^abB^	29.38 ± 1.62^bB^	29.56 ± 1.59^bB^	35.44 ± 2.63^cA^
NO_2_	Water	15.52 ± 2.03^aB^	19.26 ± 1.96^bB^	33.45 ± 0.54^cA^	56.61 ± 1.28^dB^	82.99 ± 0.64^eC^
	Ethanol	3.23 ± 0.17^aA^	13.91 ± 1.34^bA^	34.18 ± 0.70^cA^	61.87 ± 1.23^dC^	80.50 ± 1.17^eB^
	Methanol	14.64 ± 1.83^aB^	17.36 ± 0.92^bB^	43.45 ± 1.78^cC^	53.57 ± 1.09^dA^	68.96 ± 1.66^eA^

The results are expressed as mean ± SD. The significance of differences was determined by one-way analysis of variance using SPSS version 12.0. A *P *<* *0.05 indicates that the difference is significant. ^a–d^Means with different superscripts in the same row show significant difference. ^A*–*D^Means with different superscripts in the same column show significant difference. NS, not significant.

**Table 2:** Antioxidant activities of guava leaf extracts for each concentration of hydroethanolic solvent.

	Solvents	Sample concentration (*μ*g/mL)				
		50	100	250	500	1000
DPPH	30% H.E.^*^	27.06 ± 0.74^aB^	53.80 ± 2.31^bC^	92.08 ± 1.49^cB^	96.03 ± 0.04^dNS^	95.76 ± 0.00^dB^
	50% H.E.	34.66 ± 2.15^aC^	62.14 ± 1.61^bD^	95.43 ± 0.46^cC^	95.89 ± 0.11^d^	95.68 ± 0.07^cA^
	70% H.E	23.72 ± 1.01^aA^	48.82 ± 0.48^bB^	91.11 ± 2.10^cB^	95.99 ± 0.07^d^	95.83 ± 0.04^dC^
	90% H.E.	21.64 ± 0.58^aA^	41.37 ± 1.96^bA^	79.73 ± 0.53^cA^	95.96 ± 0.15^d^	95.86 ± 0.02^dC^
ABTS	30% H.E.	41.90 ± 0.74^aC^	73.44 ± 0.98^bC^	98.21 ± 0.00^cB^	98.07 ± 0.00^dB^	97.91 ± 0.04^dB^
	50% H.E.	47.48 ± 0.60^aD^	84.49 ± 0.66^bD^	98.21 ± 0.00^cB^	98.07 ± 0.00^dB^	97.79 ± 0.14^cB^
	70% H.E	38.59 ± 0.90^aB^	68.57 ± 1.08^bB^	98.62 ± 0.07^cC^	98.42 ± 0.14^dC^	97.82 ± 0.22^dB^
	90% H.E.	32.39 ± 0.45^aA^	58.53 ± 0.59^bA^	96.88 ± 0.14^cA^	97.36 ± 0.04^dA^	96.28 ± 0.36^dA^
Reducing power	30% H.E.	0.23 ± 0.00^aB^	0.34 ± 0.00^bB^	0.64 ± 0.01^cC^	1.14 ± 0.01^dC^	2.12 ± 0.01^eC^
	50% H.E.	0.26 ± 0.00^aC^	0.38 ± 0.01^bC^	0.76 ± 0.01^cD^	1.36 ± 0.01^dD^	2.39 ± 0.01^eD^
	70% H.E	0.23 ± 0.00^aB^	0.33 ± 0.01^bB^	0.61 ± 0.01^cB^	1.07 ± 0.01^dB^	2.02 ± 0.02^eB^
	90% H.E.	0.21 ± 0.00^aA^	0.29 ± 0.00^bA^	0.52 ± 0.01^cA^	0.97 ± 0.01^dA^	1.68 ± 0.01^eA^
NO	30% H.E.	42.19 ± 0.56^aB^	49.45 ± 0.94^bA^	54.86 ± 1.25^cA^	63.03 ± 1.05^dA^	68.96 ± 0.28^eB^
	50% H.E.	52.45 ± 2.45^aC^	59.96 ± 0.29^bC^	57.55 ± 0.91^cB^	66.71 ± 0.24^dB^	73.07 ± 0.00^eC^
	70% H.E	45.09 ± 3.31^aB^	58.22 ± 0.91^bC^	56.16 ± 1.41^bAB^	64.02 ± 0.49^cA^	69.10 ± 2.01^dB^
	90% H.E.	34.42 ± 4.29^aA^	55.74 ± 1.37^bB^	60.06 ± 0.94^cC^	64.49 ± 0.94^dA^	64.88 ± 1.13^eA^
NO_2_	30% H.E.	19.32 ± 1.43^aA^	34.04 ± 2.56^bAB^	67.14 ± 0.93^cB^	83.39 ± 1.41^dB^	93.52 ± 0.20^eB^
	50% H.E.	35.45 ± 2.70^aC^	47.94 ± 1.95^bC^	77.86 ± 2.48^cC^	94.11 ± 1.34^dD^	96.67 ± 0.60^dD^
	70% H.E	24.97 ± 1.47^aB^	37.22 ± 0.89^bB^	67.26 ± 0.54^cB^	86.57 ± 0.35^dC^	95.17 ± 0.20^eC^
	90% H.E.	17.79 ± 1.14^aA^	31.21 ± 1.74^bA^	59.72 ± 0.35^cA^	80.21 ± 0.35^dA^	91.99 ± 0.54^eA^

The results are expressed as mean ± SD. The significance of the differences was determined by one-way analysis of variance using SPSS version 12.0. A *P *<* *0.05 is considered significant. ^a–d^Means with different superscripts in the same row show significant difference. ^A–D^Means with different superscripts in the same column show significant difference. H.E., hydroethanolic extract; NS, not significant.

Our results are consistent with previous reports. It was shown that DPPH-^.^ and ABTS^^.^+^-scavenging activity and reducing power of guava leaves in the water extract were higher than in purely ethanol, methanol, hexane, and ethyl acetate extracts (Aktumsek et al. 2013). Furthermore, the activity of 50% hydroethanolic extract was observed to be even higher than that of the water extract (Qian and Nihorimbere 2004). It was reported that DPPH-^.^ and ABTS^^.^+^-scavenging activities were significantly correlated with the total abundance of phenolic compounds (Tayade et al. 2013). Antioxidant activity is strongly correlated with reducing power, which increased depending on the concentration and reaction time of the extracts (Kwon et al. 2013). Our results clearly suggest that the antioxidant abilities of guava leaves, such as DPPH-^.^ and ABTS^^.^+^-scavenging activity and reducing power, are closely dependent on the contents of the phenolic compounds.

### Nitric oxide (NO) radical-and nitrite (NO_2_)-scavenging activity

Nitric oxide-scavenging activity of the ethanol extract with a high content of flavonoids was significantly higher than that of the water or methanol extract, while nitrite scavenging abilities of the three solvent extracts did not differ significantly (Table 1). In the test using the mixed solvents, both nitric oxide-and nitrite-scavenging abilities were significantly higher in the 50% hydroethanolic extract that had the highest content of phenolic compounds (Table 2).

In previous studies for the flavonoid content of *Impatiens balsamina*, potato peel, sugar beet pulp, and sesame cake, the flavonoid content of purely methanol extract was higher than that of the content of water or purely ethanol extracts (Su et al. 2012). However, the flavonoid content of 50% hydroethanolic extract was higher than that of other extracts, such as water and 80% methanol extracts (Stanojević et al. 2009). Furthermore, the nitrite-scavenging activity of plum with high flavonoid content in 80% ethanol extracts was higher than that of two other kinds of plum that had low levels of flavonoid content (Kim et al. 2012).

According to this study, the water extract with a high content of phenolic compounds showed high antioxidant abilities in the DPPH-^.^ and ABTS^.+^-scavenging activity and in the reducing power assay. Ethanol extract with a high flavonoid content showed high antioxidant activities in the nitric oxide radical-and nitrite-scavenging ability assay. In the antioxidant ability tests of hydroethanolic extracts, as measured by DPPH-^.^ and ABTS^.+^-scavenging activity, reducing power, and nitric oxide and nitrite-scavenging activity, the activity of 50% hydroethanolic extract was the highest among the three different solvents and the other hydroethanolic extracts. This comparison strongly suggests that the best extraction solvent for high antioxidant efficacy of guava leaves is the 50% hydroethanolic solvent.

## Conclusion

This study intended to find the best extraction solvent for high antioxidant efficacy of guava leaves using various solvents. The phenolic compound content of water extract was higher than pure ethanol and methanol extract. Furthermore, the phenolic compound content of hydroethanolic extracts was higher than water extracts. The antioxidant activity of hydroethanolic extracts was higher than that of the water extracts and was significantly high in the 50% hydroethanolic extract that had the highest content of phenolic compounds.
